# Are parents as great as they think they are? A longitudinal study of parent–child perceived parenting discrepancies on adolescent depressive symptoms in U.S. families of Chinese origin

**DOI:** 10.1111/jora.70064

**Published:** 2025-08-15

**Authors:** Wen Wen, Cindy J. Huang, Yuan Fang, Yang Hou, Shanting Chen, Kiera Coulter, Su Yeong Kim

**Affiliations:** ^1^ Crown Family School of Social Work, Policy, and Practice The University of Chicago Chicago Illinois USA; ^2^ Department of Counseling and Clinical Psychology Teachers College Columbia University New York New York USA; ^3^ State Key Laboratory of Cognitive Science and Mental Health Institute of Psychology, Chinese Academy of Sciences Beijing China; ^4^ College of Medicine Florida State University Tallahassee Florida USA; ^5^ Department of Psychology University of Florida Gainesville Florida USA; ^6^ Population Research Center University of Texas at Austin Austin Texas USA; ^7^ Department of Human Development and Family Sciences University of Texas at Austin Austin Texas USA

**Keywords:** depressive symptoms, parent–child perceived parenting discrepancies, U.S. families of Chinese origin

## Abstract

A developmental perspective is needed to reveal the long‐lasting influence of perceived parenting discrepancies on youth depressive symptoms from early adolescence to emerging adulthood. This is particularly important for U.S. families of Chinese origin, an understudied U.S. population in research on perceived parenting discrepancies. The current study used an 8‐year longitudinal dataset of 444 youth (*M*_wave1.age_ = 13.51, SD = 0.64; 54% girls) and their mothers (*N* = 393) and fathers (*N* = 374) from U.S. families of Chinese origin to examine how convergent and divergent perceptions of parenting in early adolescence relate to depressive symptoms in emerging adulthood (*M*_wave3.age_ = 21.39, SD = 0.62). Response surface analysis revealed that when mothers, but not fathers, reported lower (versus higher) levels of hostility than adolescents in early adolescence, youth reported higher levels of depressive symptoms in emerging adulthood. The finding highlights the need for early adolescent interventions to address parent–child perceived parenting discrepancies when mothers reported less hostility than adolescents, given their lasting impact on youth depressive symptoms in U.S. families of Chinese origin.

## INTRODUCTION

Although Chinese American adolescents often show fewer externalizing symptoms and better academic performance compared with other ethnic minority groups, they may encounter greater risks for internalizing symptoms, including depressive symptoms (Castro & Rice, 2003). Parenting practices in early adolescence, a period marked by significant neuroplasticity and heightened sensitivity to environmental stimulus, can have a long‐lasting influence on Chinese American adolescents’ depressive symptoms (Kim et al., 2013). However, parenting practices may be interpreted differently by adolescents and their parents, especially during adolescence, a period characterized by rapid developmental changes in autonomy, identity formation, and family roles. The modified operations triad model (De Los Reyes & Ohannessian, 2016) provides a theoretical framework for understanding these parent–child perceived parenting discrepancies as a meaningful developmental phenomenon rather than as mere measurement error. The model proposes that discrepancies can reflect important aspects of family functioning, such as communication quality, relational attunement, and role negotiation, which may have lasting implications for youth well‐being, including depressive symptoms.

Several gaps in the previous literature are worth highlighting. First, parent–child perceived parenting discrepancies may be particularly salient but understudied in U.S. families of Chinese origin, where acculturation gaps between parents and children often result in differing cultural values and interpretations of parenting practices (Buki et al., 2003; Fung & Lau, 2010; Zhu & Chang, 2019). For example, parents may emphasize academic achievement and perceive pressuring their children as beneficial for long‐term success (Huang & Gove, 2015), while their more acculturated children may interpret such behaviors as overly controlling or harsh. These gaps in cultural values can result in discrepant perceptions, which have been linked to negative youth outcomes, including depressive symptoms (Korelitz & Garber, 2016). Second, while mothers are usually involved more in daily parent–child interactions, fathers are traditionally supposed to “*yang*” (raise) and “*jiao*” (educate) children as role models (Cao & Lin, 2019). However, research on fathers’ parenting is generally lacking in extant literature (Cabrera et al., 2018). Third, most studies examining the influence of parent–child perceived parenting discrepancies have focused on a relatively short developmental period (e.g., Córdova et al., 2016; Hou et al., 2018; Human et al., 2016; Kapetanovic & Boson, 2020), overlooking the long‐term impact across time from early adolescence to emerging adulthood. Given the developmental transitions of adolescence, short‐term findings may not fully capture lasting implications.

To address the gaps in the literature, the current study investigated the long‐term implications of perceived parenting discrepancies in early adolescence in mother–adolescent and father–adolescent dyads on adolescent depressive symptoms in emerging adulthood. Specifically, the current study adopted a longitudinal dataset across 8 years of adolescence in U.S. families of Chinese origin. Response surface analysis (RSA) was applied to directly examine how convergence and divergence patterns of mother– or father–adolescent perceptions of parenting (i.e., parental hostility and warmth) in early adolescence are related to internalizing symptoms in emerging adulthood.

## USING THE MODIFIED OPERATIONS TRIAD MODEL TO UNDERSTAND PARENT–CHILD PERCEIVED PARENTING DISCREPANCIES

Parent–child perceived parenting discrepancies are common. Prior meta‐analyses revealed a small cross‐informant correlation in perceived parenting practices between parents and adolescents (Hou et al., 2020) (Korelitz & Garber, 2016). The modified operations triad model suggests that parent–child perceived parenting discrepancies may not be simply attributable to measurement error, but are rather a meaningful indicator of family functioning (De Los Reyes & Ohannessian, 2016). According to the modified operations triad model, parent–child perceived parenting discrepancies may be adaptive or maladaptive for adolescent adjustment (De Los Reyes & Ohannessian, 2016). While parent–child perceived parenting discrepancies can reflect poor family communication and difficulties in solving conflicts, the discrepancies may also be due to the developmental changes in early adolescence, as youth are renegotiating parent–child relationships and communication styles during this transitory developmental period that is known for increases in parent–child conflict (Brković et al., 2013). One key factor that may relate to the adaptive or maladaptive nature of the perceived parenting discrepancies is its direction: whether parents (i.e., mothers or fathers) or adolescents perceive parenting more positively than the other (Wen, Chen, et al., 2023).

Based on the *Diverging Operations (*De Los Reyes & Ohannessian, 2016), when parents report more parental warmth or lower parental hostility than adolescents, it may indicate a disconnect in recognizing adolescents’ emotional needs—especially as youth seek greater autonomy in early adolescence (Zimmer‐Gembeck & Collins, 2003). Such discrepancies may also stem from parents’ socially desirable self‐presentation, which may be particularly true for Chinese parents with culturally relevant desires to “save face” (Fung & Lau, 2010; Zane & Yeh, 2002). Additionally, adolescents’ desire for more independence may lead them to perceive even well‐intentioned parenting as overly controlling (Janssen et al., 2021). Overall, such discrepancies in the diverging operations (i.e., parents perceive parenting more positively than adolescents) may reflect family functioning problems such as parents' neglect of children's needs or children's misunderstanding of parents, contributing to negative mental health outcomes for adolescents, including elevated depressive symptoms (De Los Reyes et al., 2019; De Los Reyes & Ohannessian, 2016). For example, in a 14‐day daily diary study, adolescents reported more negative daily affect when parents reported more parental warmth than adolescents (Janssen et al., 2021). It is worth noting that although less common, some adolescents report more positive or less negative parenting practices than their parents (Hou et al., 2018; Nichols & Tanner‐Smith, 2022). This may reflect greater perspective‐taking and understanding of parental intentions (Hall et al., 2021) and is associated with lower levels of adolescent depressive symptoms (Nichols & Tanner‐Smith, 2022).

In addition, *Converging Operations* indicate that congruence between parents’ and adolescents’ perceptions of high levels of positive parenting (e.g., parental warmth) or low levels of negative parenting (e.g., parental hostility) is beneficial for adolescent adjustment (e.g., low depressive symptoms) and reflects healthy family functioning (De Los Reyes & Ohannessian, 2016). However, parent–adolescent congruence at high levels of negative parenting practice (e.g., parental hostility) or low levels of positive parenting (e.g., parental warmth) may suggest familial dysfunction (De Los Reyes & Ohannessian, 2016) and may result in higher levels of adolescent depressive symptoms.

The current study focused on parental warmth and hostility perceptions to understand parent–child perceived parenting discrepancies as they capture both positive and negative parenting aspects. Parental warmth is defined as affectionate, supportive, and responsive parenting (Spinrad & Gal, 2018), while parental hostility is characterized by angry and hostile parenting behaviors (Mari & Keizer, 2021). While parental warmth reflects positive emotional responses from their parents and is related to adaptive development and positive mental health outcomes, children who experience parental hostility may develop increased internalizing problems, such as depressive symptoms (Lewis et al., 2014; Thomas et al., 2018). While European American and Chinese American youths’ ideals of general parenting practices (e.g., parental warmth and hostility) are similar, Chinese origin youths may encounter greater challenges in perceiving high levels of parental warmth or low levels of parental hostility (Wu & Chao, 2005), which may be due to the differences in the understanding, expression, and interpretation of parental warmth/hostility between parents and adolescents in immigrant families (Russell et al., 2010). For example, youths who endorse more of the U.S. mainstream culture may expect expressions of parental warmth through hugging and direct praise. In contrast, parents who endorse more traditional Chinese culture may feel less comfortable with these forms of expression, preferring to show their warmth through instrumental support, such as cooking for their children. Thus, it is crucial to understand the perception discrepancies of parental warmth and hostility among U.S. families of Chinese origin.

## LONG‐TERM EFFECTS OF PARENT–CHILD PERCEIVED PARENTING DISCREPANCIES

Despite the growing number of studies revealing the mental health consequences of parent–child perceived parenting discrepancies, most have focused on its short‐term (i.e., no more than 1 year) influence (e.g., Abar et al., 2015; Guion et al., 2009). The long‐term impact of parent–child perceived parenting discrepancies is still unclear, limiting the understanding of this topic from a developmental perspective. Ecological theories have highlighted that the influence of contextual factors on youth development depends on the timing of the contextual influence (Bronfenbrenner, 1986). Contextual factors, including parenting, can have a profound and long‐lasting influence on youth development when they occur during sensitive developmental periods (Dahl et al., 2018). That is, when parenting discrepancies are perceived during sensitive developmental periods, they can be critical in shaping youth mental health outcomes, including depressive symptoms, in the long term. Specifically, early adolescence is a particularly sensitive developmental period, as youth undergo critical brain development during this time (Casey et al., 2008), leading to plasticity and heightened sensitivity to contextual influence. That is, significant developmental changes during early adolescence may increase youth vulnerability to discrepant parenting perceptions during early adolescence in U.S. families of Chinese origin, potentially leading to variabilities in depressive symptoms years later during emerging adulthood. Moreover, parent–child perceived parenting discrepancies may be more pronounced in early adolescence. Early adolescents may have increased unsupervised time with their peers while simultaneously increasing distance from their parents, which could be a risk factor for poorer parent–child relationships (Casey et al., 2008; Fuligni & Eccles, 1993) and greater parent–child perceived parenting discrepancies. Thus, this particularly sensitive period of early adolescence may require additional parental intervention and effective parenting, necessitating parents’ and adolescents’ agreement on what constitutes positive parenting practices.

Parent–child perceived parenting discrepancies may be common during early adolescence, particularly among U.S. families of Chinese origin; however, the long‐term influence of different configurations of discrepancies (e.g., parents or children reporting higher positive parenting than the other) is poorly understood. When youth are in early adolescence, parents are often the authority figures in the family. During this developmental period, parents may believe that their own report of parenting, rather than their children's perception of parenting, is a more reliable predictor for youth mental health in the long term. This view may be rooted in the understanding that early adolescents are still undergoing socio‐cognitive development and may not yet possess the capacity to accurately judge what effective parenting is or determine the type of parenting necessary for their future development. Despite the potential appeal of this parental perspective, there is a noticeable gap in empirical research examining the long‐term impact of parent–child perceived parenting discrepancies on youth development during emerging adulthood. Thus, there is a clear need to better understand how parent–child perceived parenting discrepancies in early adolescence impact long‐term youth outcomes.

## UTILITY OF RESPONSE SURFACE ANALYSIS (RSA) IN EXAMINING PERCEIVED PARENTING DISCREPANCIES

Existing research on parent–child perceived parenting discrepancies has been conducted using different methodological frameworks (e.g., Abar et al., 2015; de Haan et al., 2018; Janssen et al., 2021; Leung & Shek, 2014; Van Petegem et al., 2020), which may contribute to inconsistent findings (de Haan et al., 2018). The difference scores and interaction approaches have been commonly used to examine parent–child perceived parenting discrepancies (Hou et al., 2020). These two methods, however, have been criticized for their lack of methodological rigor. For example, the difference score method oversimplifies the relationship between parent–child perceived parenting discrepancies and adolescent outcomes by combining four different situations (i.e., adolescents perceive more optimal parenting occurring than parents; parents perceive more optimal parenting occurring than adolescents; parents and adolescents agree that optimal parenting is occurring; parents and adolescents agree that harmful parenting is occurring) into a single score (e.g., subtracting adolescent scores from parent scores). Results from this method are ambiguous and challenging to distinguish among different situations (de Haan et al., 2018). The interaction approach moves beyond the difference scores to show the interplay of parents’ and adolescents’ perceived parenting. However, the interaction approach is not able to directly capture the degree and direction of parent–child perceived parenting discrepancies (Hou et al., 2020).

Response surface analysis (RSA) builds upon the interaction approach by estimating polynomial regressions and providing 3D visualization of findings, which is more useful than the interaction approach to directly quantify and visualize the impact of the degree of parent–child perceived parenting discrepancies on youth outcomes (Barranti et al., 2017). For example, a previous study used RSA to directly capture discrepancies in perceived family chaos between parents and adolescents and revealed that the direction of the discrepancies (i.e., significant a3 that examines the slope of the line of incongruence), rather than the degree of discrepancies (i.e., nonsignificant a4 that examines the curve of the line of incongruence), matters more for adolescent adjustment (Human et al., 2016).

Even though RSA has been gradually applied to the issue of parent–adolescent perceived parenting discrepancies (Human et al., 2016; Janssen et al., 2021), most previous studies have focused on shorter time periods, while the longitudinal effects of discrepancies on adolescent depressive symptoms among U.S. families of Chinese origin have not yet been explored. Specifically, RSA can be used to examine the associations between adolescents' and parents' perceived parenting and youth depressive symptoms 8 years later from four perspectives. For the convergent situations: (a) a1 addresses a situation when parents and children agree on parenting perceptions, whether higher parental warmth or lower parental hostility perception would relate to lower depressive symptoms 8 years later; (b) a2 addresses a situation when parents and children agree on parenting perceptions, whether their agreement on extremely high or low levels of parental warmth/hostility would relate to variabilities in depressive symptoms compared with agreement at moderate levels. For the divergent situations: (c) a3 examines whether parents perceiving more optimal parenting (i.e., higher parental warmth or lower parental hostility) than adolescents would relate to more youth's depressive symptoms 8 years later compared with adolescents who perceive more optimal parenting than parents; (d) a4 examines whether greater discrepancies, compared with agreement, between parent–adolescent perceived parenting would relate to variations in youth's depressive symptoms 8 years later.

## THE CURRENT STUDY

This study utilized a longitudinal design to investigate how convergent and divergent parent–child perceptions of parenting practices (i.e., parental warmth and parental hostility) during early adolescence influence youth depressive symptoms 8 years later in emerging adulthood among U.S. families of Chinese origin. Response surface analysis (RSA) was used to integrate mother‐ and father‐reported parenting, adolescent‐reported parenting, and adolescent depressive symptoms into a three‐dimensional surface plot. Based on the theoretical and empirical evidence above, this study addresses several limitations in existing research on parent–child perceived parenting discrepancies. First, this study aims to fill a gap in the existing literature on U.S. families of Chinese origin, guided by the modified operations triad models (De Los Reyes & Ohannessian, 2016). Second, this study examines both mother–adolescent and father–adolescent dyads, addressing the lack of research on fathers and their parenting practices in the extant literature (Janssen et al., 2021; McKinney et al., 2008). Third, while previous research has explored the longitudinal effects of parenting practices on adolescent mental health, little is known about the effects of parent–child perceived parenting discrepancies on mental health from early adolescence to emerging adulthood. This study addresses this gap by examining the long‐term effects of parent–child perceived parenting discrepancies on youth depressive symptoms in emerging adulthood.

Based on previous literature and the theoretical framework of the modified operations triad model (De Los Reyes & Ohannessian, 2016), this study focused on a1 and a3 in RSA and proposed the following two hypotheses.

Hypothesis 1 A1 in RSA (i.e., slope of the line of congruence) would be significant: Convergence in parent–adolescent perceived parental warmth at higher (vs. lower) values during early adolescence will be associated with lower youth depressive symptoms 8 years later in emerging adulthood, while convergence in parent–adolescent perceived parental hostility during early adolescence at higher (vs. lower) values will be associated with higher youth depressive symptoms 8 years later in emerging adulthood.

Hypothesis 2 A3 (i.e., slope of the line of incongruence) in RSA would be significant: The direction of the divergence matters. Parents reporting higher parental warmth or lower parental hostility than adolescents during early adolescence will relate to higher youth's depressive symptoms 8 years later in emerging adulthood, compared with parents reporting lower parental warmth or higher parental hostility than adolescents during early adolescence.

In addition, we hypothesized that a2 (i.e., curve of the line of congruence) and a4 (i.e., curve of the line of incongruence) would be nonsignificant. Specifically, we did not expect variations in youth depressive symptoms in emerging adulthood between those who agreed with parents in extreme versus moderate levels of parental warmth and hostility in early adolescence (i.e., nonsignificant a2). In addition, we expected the direction of discrepancies in parenting perceptions to matter (i.e., significant a3) rather than the degree of discrepancies regardless of direction (nonsignificant a4). Thus, we did not expect variations in youth depressive symptoms in emerging adulthood between those who had higher versus lower discrepancies in perceived parental warmth or hostility than their parents in early adolescence.

## METHOD

### Participants

The current study utilized Wave 1 and 3 data from a three‐wave longitudinal study of 444 Chinese origin families from Northern California. Waves 1 and 3 were selected to capture long‐term developmental changes over a more extended time span. In Wave 1, there were 444 middle‐school‐age youth (*M*_age_ = 13.51, SD = 0.64; 54% girls) participating with their mothers (*N* = 393; *M*_age_ = 44.00, SD = 4.79) and fathers (*N* = 374; *M*_age_ = 47.93, SD = 6.15). The median highest education level for parents was finishing high school. The median family income was in the range of $30,001 to $45,000. The majority of youth (*n* = 334, 75.4%) were born in the U.S., while only 47 fathers (12.9%) and 41 mothers (10.3%) were born in the U.S. The majority of youth in this study (*n* = 388, 87.4%) reported their parents married or living together. At Wave 3, 8 years after Wave 1, there were 324 families, including 324 youth (*M*_age_ = 21.39, SD = 0.62), 289 mothers (*M*_age_ = 51.98, SD = 4.30), and 256 fathers (*M*_age_ = 55.43, SD = 5.61). Attrition analysis showed that families with girls were more likely to continue participating in the study compared with families with boys (*χ* ^*2*^ (1) = 5.32, *p* = .02). Whether the family participated or dropped out of the study at Wave 3 was not associated with youth depressive symptoms (*t*(182.9) = 1.81, *p* = .07); it was also not associated with adolescents’ or parents’ perceived paternal warmth or hostility (*p* ranges from .05 to .99) at Wave 1.

### Procedure

Research assistants identified potential youth participants with the help of school administrators from seven middle schools. The schools were all in major metropolitan areas of Northern California. A bilingual letter in both Chinese and English was sent to potential participant families to describe the aim and procedure of the research project. Around half of the eligible families (47%) agreed to participate and provided parent consent and youth assent. Afterward, those families received a packet of paper questionnaires for mothers, fathers, and target youth.

The questionnaires were prepared in both Chinese and English. Bilingual research assistants translated question items into Chinese and then back‐translated them to English to check for consistency and accuracy. The majority of parents (71%) used the Chinese version of the questionnaires, while most youth (85%) used the English version. Participants were instructed to finish the questionnaires independently and to seal them immediately afterward. The questionnaires were then collected by research assistants from youth at schools. Among all families, 76% completed Wave 1 and were compensated $30 for their time. Wave 3 data were collected via paper surveys in the mail 8 years later, and families were compensated $130 for their participation. The procedure was reviewed and approved by the Institutional Review Board (IRB) at the University of Texas at Austin.

### Measures

Youth, mothers, and fathers reported on perceived parental warmth and parental hostility during Wave 1. Youth self‐reported depressive symptoms at Wave 1 and Wave 3.

#### Parental warmth

Parental warmth was measured using eight items adapted from measures used in the Iowa Youth and Families Project assessing parental affection (Conger et al., 1995; Ge et al., 1996). Sample items included: “Let him/her (child) know that you (parent) appreciated him/her ideas or things he/she does” and “Let him/her (child) know you (parent) really care about him/her.” Items were rated using a 7‐point Likert scale, ranging from 1 (*Never*) to 7 (*Always*). A mean score was calculated, with higher scores representing greater parental warmth. This measure demonstrated adequate internal consistency for all reporters (adolescent‐reported parental warmth for mothers: *α*_wave 1_ = .91; adolescent‐reported parental warmth for fathers: *α*_wave 1_ = .92; mothers' self‐report: *α*_wave 1_ = .88; fathers' self‐report: *α*_wave 1_ = .89).

#### Parental hostility

Parental hostility was measured using eight items adapted from measures used in the Iowa Youth and Families Project assessing parental hostility (Conger et al., 1995; Ge et al., 1996). Sample items included: “Shout or yell at him/her (child) because you (parent) were mad at him/her” and “Hit, push, grab, or shove him/her (child).” Items were rated using a 7‐point Likert scale, ranging from 1 (*Never*) to 7 (*Always*). A mean score was calculated, with higher scores representing greater parental hostility. This measure demonstrated adequate internal consistency for all reporters (adolescent‐reported parental hostility for mothers: *α*_wave 1_ = .83; adolescent‐reported parental hostility for fathers: *α*_wave 1_ = .84; mothers' self‐report: *α*_wave 1_ = .79; fathers' self‐report: *α*_wave 1_ = .82).

#### Youth depressive symptoms

Youth depressive symptoms were measured using 20 items from the Center for Epidemiologic Studies of Depression Scale (CES‐D; Radloff, 1977). Sample items included: “Did not feel like eating; appetite was poor” and “Had trouble keeping the mind focused on what one was doing.” Items were rated using a 4‐point Likert scale, ranging from 0 (*Rarely or none of the time*) to 3 (*Most or all of the time*). A mean score was calculated, with higher scores representing more depressive symptoms. This measure demonstrated adequate internal consistency (*α*_wave 1_ = .87; *α*_wave 3_ = .90).

### Covariates

Adolescents’ age, gender (i.e., self‐reported female or male), and nativity (i.e., born in the U.S. or not) were included as covariates, as these variables have been shown to be associated with depressive symptoms (Akhtar‐Danesh & Landeen, 2007). Maternal or paternal highest education level was included as a covariate in models including mothers or fathers, respectively. Adolescent Wave 1 depressive symptoms were also included as a covariate to account for baseline depressive symptoms.

### Data analysis

Analyses were conducted in two steps. First, descriptive statistics and Pearson correlations were conducted in SPSS 22.0 to provide basic information about and to understand associations between variables. Second, response surface analyses (RSA) were conducted in R using the R package “RSA.” (Schönbrodt & Humberg, 2023). There were four RSA models estimated to investigate how convergent and divergent mean‐centered perceptions of maternal/paternal warmth and hostility at Wave 1 were associated with adolescent depressive symptoms 8 years later in emerging adulthood at Wave 3 after controlling for demographic variables and Wave 1 depressive symptoms. There were in total four RSA models estimated to examine maternal and paternal hostility and warmth, respectively. RSA coefficients were evaluated to answer four questions: whether convergence at high levels of the measured parenting practice was associated with more youth depressive symptoms 8 years later in emerging adulthood compared with convergence at low levels (coefficient a1); whether convergence at extreme values (i.e., high or low levels) of the measured parenting practice was associated with more youth depressive symptoms 8 years later in emerging adulthood compared with convergence at median levels (coefficient a2); whether youth report of higher levels of the measured parenting practice was related to more youth depressive symptoms 8 years later in emerging adulthood compared with parent report of higher levels of the measured parenting practice (coefficient a3); and whether divergent reports of the measured parenting practice were associated with more youth depressive symptoms 8 years later in emerging adulthood compared with convergent reports (coefficient a4). Full information maximum likelihood (FIML) was used to handle missing data.

To test the robustness of the examined associations, two sets of sensitivity analyses were conducted. The first set of analyses includes maternal (or paternal) parenting perceptions as a covariate in the paternal (or maternal) RSA model to examine whether the effects were robust to the inclusion of the other parent's parenting. The second set is to investigate whether youth depressive symptoms in emerging adulthood were directly driven by adolescents’ or parents’ perceived parenting in early adolescence. Linear regression analyses were conducted to examine the longitudinal link from adolescents,’ mothers', and fathers' perceived parenting (parental warmth and hostility) in early adolescence to adolescents’ depressive symptoms in emerging adulthood after controlling for all covariates. The main analysis with this set of sensitivity analyses can better reveal whether the parent–child perceived parenting discrepancies or each family member's perception of parenting individually can influence youth depressive symptoms in the long run.

## RESULTS

Descriptive information and bivariate correlations for study variables are displayed in Table 1. Youth who reported more maternal warmth at Wave 1 reported fewer depressive symptoms in emerging adulthood at Wave 3. Youth who reported more maternal hostility at Wave 1 reported more depressive symptoms in emerging adulthood at Wave 3. Youth‐reported paternal warmth and hostility for fathers, as well as mother‐ or father‐reported parental warmth and hostility at Wave 1, were not associated with Wave 3 youth depressive symptoms in emerging adulthood.

**TABLE 1:** Descriptive information and correlation between study variables.

	1	2	3	4	5	6	7	8	9	10	11	12	13	14	*M*	SD
1. Youth Wave 3 age	‐														21.391	0.620
2. Youth nativity (0 = U.S.)	.071	‐													0.246	‐
3. Youth gender (0 = boys)	−.023	.007	‐												0.540	‐
4. Maternal highest education	.027	−.142**	−.023	‐											5.862	1.718
5. Paternal highest education	−.051	−.047	.016	.621***	‐										5.927	1.817
6. Youth Wave 1 maternal warmth	−.056	.006	.047	.161**	.188***	‐									4.972	1.331
7. Youth Wave 1 maternal hostility	.073	.019	.034	.041	.024	−.327***	‐								2.747	1.114
8. Youth Wave 1 paternal warmth	−.132*	−.016	.024	.180***	.177**	.742***	−.165**	‐							4.715	1.432
9. Youth Wave 1 paternal hostility	.097	.033	−.075	.097	.111*	−.071	.586***	−.249***	‐						2.594	1.116
10. Mother Wave 1 maternal warmth	−.016	−.059	.110*	.163**	.071	.224***	−.093	.247***	−.059	‐					5.835	0.888
11. Mother Wave 1 maternal hostility	.033	.086	−.063	−.021	.009	−.137**	.270***	−.132**	.189***	−.243***	‐				2.702	0.848
12. Father Wave 1 paternal warmth	−.074	−.077	.134**	.073	.010	.108*	−.120*	.127*	−.054	.439***	−.183**	‐			5.628	0.969
13. Father Wave 1 paternal hostility	.016	.074	−.050	.006	.028	−.024	.070	−.070	.164**	−.099	.394***	−.145**	‐		2.545	0.845
14. Youth Wave 1 depressive symptoms	.068	.040	.063	−.059	−.153**	−.353***	.302***	−.341***	.228***	−.047	.171**	.043	.052	‐	0.642	0.421
15. Youth Wave 3 depressive symptoms	−.026	.079	.192**	−.036	−.055	−.133*	.148**	−.067	.064	.041	−.032	.032	.026	.266***	0.629	0.451

* *p* < .05.

** *p* < .01.

*** *p* < .001.

**FIGURE 1:**
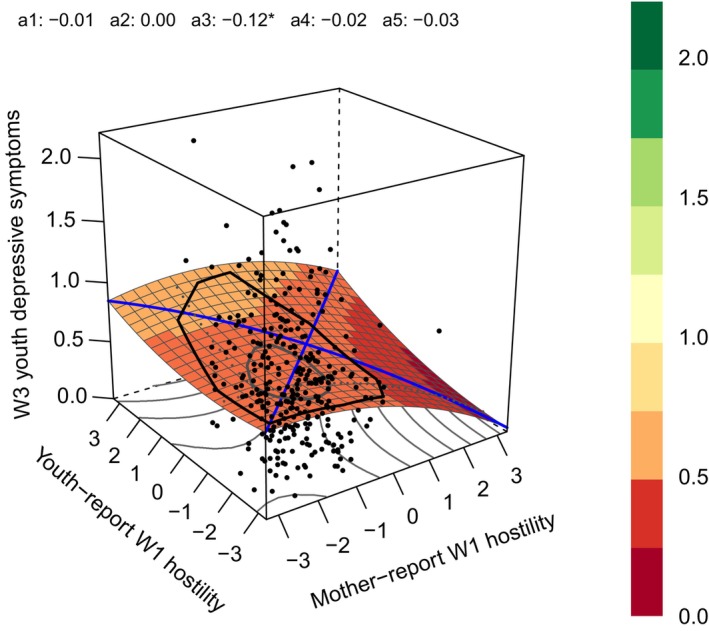
Response surface analysis plot for mother–youth reported maternal hostility at Wave 1 and youth depressive symptoms at Wave 3. W, Wave. A1 is the slope of and a2 is the curve of the line of the congruence (i.e. when mother and youth reported convergent levels of hostility); a3 is the slope of and a4 is the curve of the line of the incongruence (i.e. when mothers and youth reported divergent levels of hostility); a5 is not interpreted in RSA. *p < .05.

Results of the RSA are given in Table 2. Contrary to Hypothesis 1, a1 was not significant in all models (Figure 1), suggesting that adolescents’ and parents’ convergence in higher or lower levels of parental warmth or hostility in early adolescence was not associated with youth depressive symptoms in emerging adulthood. In partial support of Hypothesis 2, after control variables were accounted for, mothers reporting lower levels of maternal hostility than adolescents in early adolescence were associated with higher youth depressive symptoms at Wave 3 in emerging adulthood (i.e., the upper left corner in Figure 1), compared with situations when mothers reported higher levels of maternal hostility than adolescents (i.e., the lower right corner in Figure 1; *a3* = −0.123, SE = 0.057, *p* = .031). The same pattern was shown for maternal warmth with marginal significance for a3 (*a3* = 0.180, SE = 0.108, *p* = .097). Specifically, there was a trend that mothers reporting higher levels of maternal warmth than adolescents in early adolescence were associated with higher youth depressive symptoms at Wave 3 in emerging adulthood, compared with when youth reported higher levels of maternal warmth than mothers.

**TABLE 2:** Youth‐parent convergent and divergent perceptions of parental warmth and hostility influencing youth depressive symptoms 8 years later.

Positive parenting predictors	Polynomial regression coefficients						RSA coefficients			
	Intercept	Parent report	Youth report	Parent report^2^	Parent report X youth report	Youth report^2^	a1	a2	a3	a4
	(SE)	(SE)	(SE)	(SE)	(SE)	(SE)	(SE)	(SE)	(SE)	(SE)
Maternal warmth	0.534***	0.138	−0.042	−0.034	0.008	0.003	0.096	−0.023	0.180^†^	−0.039
	(0.059)	(0.073)	(0.047)	(0.025)	(0.022)	(0.013)	(0.058)	(0.021)	(0.108)	(0.045)
Maternal hostility	0.607***	−0.068	0.055	−0.017	0.009	0.011	−0.013	0.002	−0.123*	−0.015
	(0.059)	(0.049)	(0.043)	(0.018)	(0.027)	(0.017)	(0.072)	(0.029)	(0.057)	(0.043)
Paternal warmth	0.606***	0.067	−0.027	−0.029	0.022	0.004	0.039	−0.003	0.094	−0.046^†^
	(0.049)	(0.057)	(0.033)	(0.020)	(0.017)	(0.010)	(0.069)	(0.028)	(0.062)	(0.027)
Paternal hostility	0.710***	0.081	0.016	0.017	0.032	−0.018	0.096	0.030	0.065	−0.033
	(0.076)	(0.062)	(0.046)	(0.016)	(0.029)	(0.014)	(0.094)	(0.034)	(0.056)	(0.037)

*Note*: Youth age at Wave 3, youth gender, nativity, parental highest education level, and Wave 1 youth depressive symptoms were controlled in the models.

^†^ *p* < .10.

* *p* < .05.

*** *p* < .001.

The results of the first sensitivity analyses showed similar results as the main analysis with the same significance (Table S1). Specifically, mothers reporting lower (vs. higher) levels of hostility than adolescents were associated with youth's higher levels of depressive symptoms 8 years later. The second set of analyses showed that parent–adolescent perceived parenting discrepancies, rather than the levels of parenting perceived by either parents or adolescents, were associated with youth depressive symptoms 8 years later. Specifically, neither adolescents’, mothers’, nor fathers’ perceived parental warmth or hostility in early adolescence was directly related to youth depressive symptoms in emerging adulthood after controlling for the same covariates as in the RSA model (Table S2). This suggests the value of the RSA method in capturing parenting perceptions among parent–adolescent dyads within the family system as opposed to examining parents’ and adolescents’ perceptions independently.

## DISCUSSION

This study examined the long‐term effects of perceived parenting discrepancies between parents and their early‐adolescent children on youth depressive symptoms in emerging adulthood among U.S. families of Chinese origin. Using RSA to capture parent–child perceived parenting discrepancies, our findings indicated that one type of parent–child perceived parenting discrepancies (i.e., mothers reporting less negative or more positive parenting than adolescents) can be particularly detrimental to youth depressive symptoms in emerging adulthood, after controlling for depressive symptoms in early adolescence. On the other hand, when youth and parents were in agreement on parenting, whether they perceived parenting as more positive or negative, congruence in parenting perceptions did not relate to youth's depressive symptoms 8 years later. In addition, as expected, neither agreeing on more extreme (versus moderate) levels of parenting perceptions nor higher degrees of discrepancies (versus agreement) on parent–child perceived parenting was related to variations in youth depressive symptoms. The findings highlight the importance of going beyond the parenting perceived by each family member to consider the parenting experiences from both parents’ and adolescents’ perspectives together. Also, it is worth noting that although youth may leave home for college or work and have less exposure to parents and their parenting practices in emerging adulthood (Montgomery & Côté, 2003), parenting experiences can have a long‐term influence on youth depressive symptoms.

Based on the modified operations triad model (De Los Reyes & Ohannessian, 2016), one major contribution of the current study is revealing the long‐term influence of early‐adolescence parent–child perceived parenting discrepancies on youth depressive symptoms among U.S. families of Chinese origin. The study revealed that one direction of the discrepancy (i.e., parents reported more positive or less negative parenting compared with adolescents) can be a risk factor suggesting family functioning problems, reflected by youth's heightened depressive symptoms 8 years later. Such patterns may reflect deeper issues in family functioning, including poor parent–child communication, low parental attunement, or cultural norms that discourage emotional expressiveness (De Los Reyes et al., 2019; De Los Reyes & Ohannessian, 2016). Those underlying family functioning problems can contribute to youth depressive symptoms in the long term (Huang et al., 2022). On the other hand, the other direction (i.e., adolescents reporting higher positive or lower negative parenting compared with parents) could be a promotive factor and related to lower levels of depressive symptoms. Specifically, aligning with the modified operations triad model (De Los Reyes & Ohannessian, 2016), adolescents who report less hostility than their mothers may be less sensitive to maternal hostility or have a greater understanding of parental behaviors. This understanding or acceptance may contribute to improved self‐acceptance and emotion regulation, thereby protecting youth from depressive symptoms in emerging adulthood (Gonçalves et al., 2019). This finding was consistent with previous studies (e.g., Leung et al., 2016; Wen, Chen, et al., 2023) and the modified operations triad model (De Los Reyes & Ohannessian, 2016), which suggests that parent–child perceived parenting discrepancies can be adaptive or maladaptive.

A similar pattern was observed for maternal warmth, although the association was only marginally significant. Given the marginal significance of this finding, interpretations regarding warmth discrepancies should be made with caution. The difference in findings between maternal hostility versus warmth may be attributed to adolescents’ heightened sensitivity to negative parenting behaviors compared with positive ones. A meta‐analysis has shown that parental hostility exerts the strongest influence on youth depressive symptoms among various parenting dimensions (McLeod et al., 2007). Such negative bias can be deeply rooted in human survival: being more sensitive to potential threats or harm (e.g., parental anger or rejection) may have conferred evolutionary advantages (Baumeister et al., 2001). Consequently, negative interpersonal experiences like hostile parenting may have a more pronounced and lasting impact on emotional and psychological development than the absence of positive parenting behaviors.

The results highlight the importance for parents to be aware of the potential discrepancies in early adolescence, particularly the possibility of overestimating positive or underestimating negative parenting. However, even when parents recognize their children's dissatisfaction, they may continue with established practices due to the difficulty of behavioral change or beliefs that adolescents lack the maturity to evaluate parenting accurately. As a result, despite being aware of perceived parenting discrepancies, these parents may persist in their practices (Wen, Sim, et al., 2023), believing that their approach is optimal and best. A key insight from this study is that such “misunderstandings,” especially when adolescents perceive greater hostility than mothers do, may not resolve over time and can contribute to elevated depressive symptoms in emerging adulthood.

In terms of the *converging operations* (De Los Reyes & Ohannessian, 2016), contrary to hypothesis 1, when youth and parents were in agreement on parenting perceptions, levels of parental warmth or hostility in early adolescence were not associated with youth depressive symptoms in emerging adulthood. This is inconsistent with the modified operational triad model (De Los Reyes & Ohannessian, 2016), which argues that adolescents’ and parents’ convergent perceptions in high levels of positive or low levels of negative parenting can benefit youth development compared with convergence in low levels of positive or high levels of negative parenting. One possible explanation is the extended time frame in this study (i.e., 8 years) whereas prior studies supporting the model often examined shorter spans (Hou et al., 2018; Human et al., 2016; Janssen et al., 2021; Nichols & Tanner‐Smith, 2022). It is possible that when youth and parents agree on parenting in early adolescence, even if the parenting is highly negative or minimally positive, adolescents may adapt by developing coping mechanisms (Williams & McGillicuddy‐De Lisi, 1999), leading to relatively low levels of depressive symptoms in emerging adulthood.

In summary, the results suggest that in the long run across 8 years, youth may be able to cope with negative or less positive early‐adolescence parenting experiences if such parenting is a consensus in the family. However, the discrepancies in which mothers underreport hostility or overreport warmth may be particularly challenging that bring extra burden and distress for adolescents, resulting in heightened depressive symptoms in emerging adulthood. Moreover, revealing such a phenomenon among U.S. families of Chinese origin can be particularly intriguing. While traditional Chinese culture emphasizes filial piety, including the values of respecting, obeying, and caring for parents and maintaining family harmony (Russell et al., 2010), the U.S. mainstream culture values independence and autonomy from parents. Different interpretations and discrepancies in perceptions of parental hostility may arise when parents endorse more Chinese traditional culture and youth endorse more U.S. mainstream cultures (Buki et al., 2003). The findings highlight the importance of identifying those parent–child perceived parenting discrepancies in early adolescence and implementing interventions to reduce such discrepancies when adolescents perceive more hostility than mothers.

The findings are mainly for mothers, and father–child perceived parenting discrepancies seem to have little influence on youth internalizing symptoms across 8 years. Few previous studies regarding discrepancies in parenting have focused on fathers. As one of the few studies considering both parents’ perceptions separately, the current study reveals the potential differences in mother–adolescent versus father–adolescent parenting perceptions and their influence on youth development.

In the current sample, 258 adolescents reported mothers as the primary caregiver in early adolescence, compared with 38 adolescents who reported fathers. In many families, mothers often assume the role of primary caregivers and spend more time with their children during early adolescence. In Chinese culture, mothers are frequently perceived as the primary source of emotional support (Lamb, 2013), which enhances the likelihood of daily communication and interaction between mothers and their adolescent children. Consequently, parent–child perceived parenting discrepancies, where mothers report more positive or less negative parenting than their early‐adolescence children, may indicate underlying family functioning issues. Those may include poor communication quality and difficulty in solving conflicts, despite the extensive opportunities for mother–child communication. On the other hand, fathers in Chinese families are often less emotionally expressive (Lamb, 2013), which may result in less frequent emotional exchange and communication with adolescents. As a result, father–child perceived parenting discrepancies may be anticipated, rather than reflecting family dysfunction. In summary, the findings suggest potential different dynamics in mother–adolescent versus father–adolescent interactions, underscoring the importance of including both parents in research on parent–child perceived parenting discrepancies. However, despite the sensitivity analysis showing the robustness of the finding after controlling for the other parent's parenting, the comparison needs to be made with caution, as there is no direct comparison between mother–adolescent versus father–adolescent perceived parenting discrepancies in the same models and the relatively smaller sample size of fathers.

Despite the contributions, this study had some limitations. Although investigating the nuances within a homogeneous sample is a strength, the results may not be generalizable outside of U.S. families of Chinese origin. Second, this study only focuses on internalizing symptoms, though perceived parenting discrepancies may also affect other domains such as externalizing behaviors, academic outcomes, and physiological health. Future research should examine multiple developmental outcomes to provide a more comprehensive understanding. Also, the current study focuses on examining the outcomes of parent–child perceived parenting discrepancies; as such, the precursors that lead to discrepancies are not clear. Future studies can consider the impact of sociocultural factors, such as adolescents’ and parents’ acculturation levels, on parent–child perceived parenting discrepancies. For example, in Chinese culture, parenting practices may be rooted in Confucian thought, highlighting the parental role of teaching or educating children to behave in culturally approved and desirable ways, which may be viewed as “controlling” and associated with “tiger parenting” from a Western perspective (Bempechat et al., 2022; Kim et al., 2013). Thus, parents and children with varying levels of endorsement of Chinese and U.S. culture may view these culturally embedded parenting practices differently, resulting in parent–child perceived parenting discrepancies. Lastly, although the current study adopted a longitudinal dataset across 8 years and controlled for baseline depressive symptoms, the study design remains correlational without manipulation of the predictors.

In summary, while previous studies have focused on a relatively short‐term influence of perceived parenting discrepancies on youth outcomes, the current study examines this association spanning 8 years from early adolescence to emerging adulthood. By focusing on U.S. families of Chinese origin, the current longitudinal study advances the understanding of the impact of perceived parenting discrepancies among this understudied population. With significant slopes of the line of incongruence in RSA, the study reveals that mothers reporting lower levels of parental hostility than adolescents in early adolescence were a risk factor for youths’ heightened depressive symptoms in emerging adulthood, while adolescents reporting lower levels of parental hostility than mothers may be a promotive factor and related to lower depressive symptoms in emerging adulthood. The findings highlight the need to examine parenting practices from both parents' and adolescents' perspectives to comprehensively capture family functioning related to parenting. Moreover, the findings speak to the need for interventions in early adolescence to identify families where mothers underreport hostility than adolescents and help enhance mother–adolescent communication qualities and reduce maternal hostility behaviors among those families.

## AUTHOR CONTRIBUTIONS

Wen Wen: Conceptualization; methodology; visualization; formal analysis; writing—original draft; writing—review & editing. Cindy J. Huang: Writing—original draft; writing—review & editing. Yuan Fang: Validation; writing—original draft; writing—review and editing. Yang Hou: Methodology; writing—review and editing. Shanting Chen: Validation; writing—review and editing. Kiera Coulter: Validation; writing—review and editing. Su Yeong Kim: Supervision; investigation; resources; funding acquisition; project administration; writing—review and editing. All authors read and approved the final manuscript and agreed on the authorship order.

## FUNDING INFORMATION

This research was supported through awards to Su Yeong Kim from (1) National Science Foundation, Division of Behavioral and Cognitive Sciences, 1651128 and 0956123; (2) National Institute on Minority Health and Health Disparities 1R21MD012706‐01A1 and 3R21MD‐012706‐02S1; (3) Eunice Kennedy Shriver National Institute of Child Health and Human Development 5R03HD060045‐02; (4) Russell Sage Foundation, 2699; (5) Spencer Foundation, 10023427; (6) Hogg Foundation for Mental Health JRG‐102; (7) Office of the Vice President for Research and Creative Grant and Special Research Grant from the University of Texas at Austin; (8) College of Natural Sciences Catalyst Grant from the University of Texas at Austin; and (9) Eunice Kennedy Shriver National Institute of Child Health and Human Development 2P2CHD042849‐21A1 and 2T32HD007081‐46A1 grants awarded to the Population Research Center at The University of Texas at Austin. This research was also supported through an award to Kiera M. Coulter from the National Institute on Minority Health and Health Disparities 5K99MD019319‐02. No potential competing interest was reported by the authors.

## CONFLICT OF INTEREST STATEMENT

The authors declare no competing interests.

## ETHICAL APPROVAL

All procedures performed in studies involving human participants were in accordance with the ethical standards of the institutional and/or national research committee and with the 1964 Helsinki declaration and its later amendments or comparable ethical standards.

## INFORMED CONSENT

Informed consent was obtained from all individual participants included in the study.

## Supporting information


Tables S1–S2.

## Data Availability

The data that support the findings of this study are available on request from the corresponding author. The data are not publicly available due to privacy or ethical restrictions.
