# Supplementary material for: Are parents as great as they think they are? A longitudinal study of parent–child perceived parenting discrepancies on adolescent depressive symptoms in U.S. families of Chinese origin
Source: J Res Adolesc. 2025 Aug 15;35(3):e70064. doi: 10.1111/jora.70064 (PMC12356167; doi:10.1111/jora.70064)
Supplement: Supplementary file 1 — Tables S1–S2. [file JORA-35-0-s001.docx]

| **Table S1** |  |  |  |  |  |  |  |  |  |  |  |
| --- | --- | --- | --- | --- | --- | --- | --- | --- | --- | --- | --- |
| *Youth-parent convergent and divergent perceptions of parental warmth and hostility influencing youth depressive symptoms eight years later* | | | | | | | | | | |  |
|  | Polynomial regression coefficients | | | | | | RSA coefficients | | | |  |
|  | Intercept | Parent report | Youth report | Parent report^2^ | Parent report X youth report | Youth report^2^ | a1 | a2 | a3 | a4 |  |
|  | (*SE*) | (*SE*) | (*SE*) | (*SE*) | (*SE*) | (*SE*) | (*SE*) | (*SE*) | (*SE*) | (*SE*) |  |
| Maternal warmth | 0.58 | 0.133 | -0.081 | -0.035 | 0.013 | 0.003 | 0.053 | -0.02 | 0.214 | -0.045 |  |
|  | 0.07 | 0.074 | 0.056 | 0.026 | 0.022 | 0.013 | 0.067 | 0.021 | 0.114 | -0.038 |  |
| Maternal hostility | 0.599 | -0.091 | 0.068 | -0.021 | 0.009 | 0.011 | -0.023 | -0.001 | -0.159^*^ | -0.019 |  |
|  | 0.066 | 0.053 | 0.045 | 0.018 | 0.026 | 0.018 | 0.075 | 0.029 | 0.065 | 0.044 |  |
| Paternal warmth | 0.562 | 0.079 | 0.006 | -0.033 | 0.018 | 0.012 | 0.086 | -0.002 | 0.073 | -0.039 |  |
|  | 0.052 | 0.058 | 0.037 | 0.021 | 0.017 | 0.01 | 0.068 | 0.028 | 0.07 | 0.029 |  |
| Paternal hostility | 0.721 | 0.116 | 0.01 | 0.022 | 0.03 | -0.012 | 0.126 | 0.04 | 0.107 | -0.02 |  |
|  | 0.083 | 0.064 | 0.048 | 0.015 | 0.028 | 0.014 | 0.097 | 0.033 | 0.059 | 0.036 |  |
| *Note.* Youth age at Wave 3, youth gender, nativity, parental highest education level, Wave 1 youth depressive symptoms, and perceptions of the other parent's parenting were controlled in the models. | | | | | | | | | | |  |
|  |  |  |  |  |  |  |  |  |  |  |  |
| * *p* < .05;***p* < .01; *** *p* < .001. | | | | | | | | | | |  |

| **Table S2** |  |  |  |  |  |  |  |  |
| --- | --- | --- | --- | --- | --- | --- | --- | --- |
| *Linear regression between Wave 1 adolescents' and parents' parenting perception and Wave 3 youth depressive symptoms* | | | | | | | | |
|  | Maternal Warmth | | Maternal Hostility | | Paternal Warmth | | Paternal Hostility | |
|  | *b* (*SE*) | *p* | *b* (*SE*) | *p* | *b* (*SE*) | *p* | *b* (*SE*) | *p* |
| W1 adolescent report parenting | - 0.016 (0.023) | .492 | 0.021 (0.026) | .422 | 0.001 (0.021) | .960 | -0.012 (0.026) | .654 |
| W1 parent report parenting | 0.040 (0.030) | .188 | -0.048 (0.031) | .130 | -0.002 (0.029) | .937 | 0.003 (0.032) | .923 |
| W1 depressive symptoms | 0.293 (0.071) | <.001 | 0.295 (0.072) | <.001 | 0.262 (0.074) | <.001 | 0.269 (0.073) | <.001 |
| W3 Youth age | -0.044 (0.042) | .297 | -0.044 (0.041) | .288 | -0.021 (0.045) | .647 | -0.019 (0.045) | .675 |
| Youth gender (0 = male) | 0.150 (0.052) | .004 | 0.151 (0.052) | .004 | 0.188 (0.056) | <.001 | 0.187 (0.056) | <.001 |
| Youth nativity (0 = US) | 0.070 (0.060) | .243 | 0.072 (0.060) | .233 | 0.067 (0.063) | .288 | 0.067 (0.063) | .286 |
| Maternal education | -0.004 (0.016) | .791 | -0.004 (0.016) | .793 | -0.006 (0.016) | .728 | -0.004 (0.016) | .783 |
| *Note.* W = Wave. |  |  |  |  |  |  |  |  |
